# Traditional Chinese medicine Tongxie Yaofang treating irritable bowel syndrome with diarrhea and type 2 diabetes mellitus in rats with liver-depression and spleen-deficiency: A preliminary study

**DOI:** 10.3389/fnut.2022.968930

**Published:** 2022-11-10

**Authors:** Weidong Xu, Zhiyi Zhang, Ye Lu, Mengxi Li, Jiayao Li, Wenhua Tao

**Affiliations:** ^1^Department of Traditional Chinese Medicine, Affiliated Hospital of Jiangsu University, Zhenjiang, China; ^2^School of Pharmacy, Jiangsu University, Zhenjiang, China

**Keywords:** TXYF, inflammation, D-IBS, T2DM, RAGE

## Abstract

Tongxie Yaofang (TXYF), a Traditional Chinese Medicine (TCM) with four components as follows: *Rhizoma Atractylodis Macrocephalae* (baizhu), *Radix Paeoniae Alba* (baishao), *Pericarpium Citri Reticulatae* (chenpi) and *Radix Saposhnikovia Divaricata* (fangfeng), benefits irritable bowel syndrome (IBS). Nonetheless, proofs of this formula ameliorating D-IBS and T2DM are required. This research aimed at investigating the efficacy of TXYF in treating inflammation in rats with D-IBS and T2DM using animal models. In this study, gavage with high-fat diet, fasciculation, and senna was given to develop rat models with target diseases. To determine intestinal inflammations, major inflammatory factors, and intestinal permeability proteins, H&E staining, ELISA, and immunohistochemistry methods were employed, respectively. This study also utilized Western blot to discover potential inflammatory targets. Results of this research illustrates that TXYF treatment reduced the level of TNF-α, IL-1β, and IL-6, and raised the IL-10 concentration in liver-depressed spleende ficient rats with D-IBS and T2DM, indicating controlled inflammatory reactions. Staining analysis also showed improved disease states of animal models. Furthermore, efficient rebounds of claudin-1, an intestinal permeability-associated protein, were detected. Moreover, TXYF may treat D-IBS and T2DM in rats *via* the rage pathway.

## Introduction

Irritable bowel syndrome (IBS) is a digestive illness marked by recurrent stomach pain or discomfort, as well as changes in bowel habits and stool patterns. IBS is a worldwide problem, with prevalence rates ranging from 5 to 15% in Western countries (1), and 5–10% in Asia (2, 3). According to an assessment by Peking Union Medical College Hospital, IBS is one of the most common ailments in urban China. Meanwhile, patients with IBS account for 34.3% in outpatient gastrointestinal clinic appointments (4). Among all types of IBS, irritable bowel syndrome with diarrhea (D-IBS) that can cause long-term symptoms and significant impacts on both physical and mental health, is difficult to be cured and may easily recur. However, the etiology of D-IBS remains unclear.

Diabetes mellitus is a metabolic disorder marked by elevated blood sugar levels, whereas chronic hyperglycemia can cause vision problems, cardiovascular and renal organ damage, and even organ failure. Type 2 diabetes mellitus (T2DM) with risk factors as listed: age, gender, obesity, dyslipidemia, genetic abnormalities, and pre-diabetes (such as IFG and IGT), takes about 90% of all diabetes. According to the International Diabetes Federation, 380 million people in the world will have diabetes by 2025 (5). Besides, diabetic complications are growingly common in T2DM patients and may have substantial influences on their lives (6).

Nevertheless, the prevalence of pre-diabetes was considerably higher in IBS patients than in control groups, indicating IBS as a risk factor for diabetes. In a previous random controlled study on 201 D-IBS patients and 220 healthy volunteers at Yangzhou University Affiliated Hospital, the incidence of diabetes was significantly higher in the IBS group, especially in patients with a longer disease course, and IBS may be related to diabetes (7). Hence, detecting risk factors of diabetes in time is critical for its prevention.

Intestinal mucosa immunity is an important system in intestinal mechanism, and its disequilibration may lead to chronic inflammation and be connected with D-IBS. Although most patients with D-IBS show no pathology change in routine histological examinations, a quantitative histological study and electron microscopic ultrastructural analysis have detected the enlarged space among epithelial cells and cytoskeletal cohesion in the intestinal mucosa of D-IBS patients (8), which may result from aberrant synthesis of tight junction protein 1 (claudin-1) that plays a significant role in regulating the osmotic pressure of intestinal mucosa. Reduced claudin-1 level in intestinal mucosa may increase intestinal osmolality (9), vice versa (10), as low-grade inflammation may cause bacterial or protease-induced breakdown (11). Meanwhile, morphological changes of intestinal mucosa that frequently accompanied by changes in immune cells and intestinal endocrine cells, may alter its permeability (12–14), which could allow more lipopolysaccharides and other inflammatory factors entering into the circulatory system and improve the absorption of endotoxins in intestines, resulting in chronic systemic inflammatory response, insulin resistance, and even diabetes (15).

Inflammatory factors are important components in immunological and inflammatory responses. Interleukin (IL) 10 is the first discovered anti-infection immunomodulatory factor in mice by inhibiting IL-1, IL-2, IL-3, IL-12, chemokines, TNF-α, and Th1 cytokines from macrophages (16). Nonetheless, the expression of IL-10 can be aberrant in patients with autoimmune diseases (17, 18), including D-IBS that may lower the IL-10 level in plasma (19, 20). Besides, high serum HbA1c expression in patients with T2DM may cause a decrease of IL-10, therefore high IL-10 grade may be related to hyperglycemia (21). Furthermore, reduced IL-10 production can interfere the down-regulate of inflammatory response in intestinal mucosa in patients with D-IBS and T2DM, which may lead to chronic low-grade inflammation. Furthermore, inflammatory agents and chemokines can impair the function of cellular carriers, influence intestinal permeability, and exacerbate the inflammatory response (11).

TXYF was initially recorded by Zhu in *Dan-Xi-Xin-Fa* as “in treating unpleasant diarrhea, prepare 3 taels of *Rhizoma Atractylodis Macrocephalae* (baizhu), 2 taels of stir-baked *Radix Paeoniae Alba* (baishao), 2.5 taels of dried *Pericarpium Citri Reticulatae* (chenpi) and 1 tael of *Radix Saposhnikovia Divaricata* (fangfeng).” According to traditional Chinese medicine (TCM), this formula can treat diarrhea with pain that results from liver-depression and spleen-deficiency by balancing and relieving liver and spleen, and dispelling dampness, including acute enteritis, chronic colitis, and IBS. TXYF on patients with D-IBS illustrated efficacy to a certain extent in some studies (22). Plants like barley are rich in bioactive phytochemicals that may exhibit an anti-obesity effect (23). Meanwhile, it may also decrease the blood glucose level by inhibiting glucosidase and dipeptidyl peptidase-IV (DPP-IV), which can effectively degrade glucagon-like peptide 1 (GLP-1) and gastric inhibitory peptide (GIP) that stimulating the production of insulin (24). As a result, TXYF has the potential to be employed in the future to prevent and control obesity-related disorders.

Hence, it’s reasonable to explore the efficacy and its mechanism of TXYF in treating D-IBS and T2DM with rat models.

## Materials and methods

### Drugs and formulations

*Rhizoma Atractylodis Macrocephalae* (baizhu), *Radix Paeoniae Alba* (baishao), *Pericarpium Citri Reticulatae* (chenpi) and *Radix Saposhnikovia Divaricata* (fangfeng) (Huahong Pharmaceutical Company, China) in this study were identified by Prof. Ouyang Zhen, School of Pharmacy, Jiangsu University.

According to Chinese Pharmacopoeia, 6 g of baizhu, 4 g of baishao, 3 g of chenpi, and 2 g of fangfeng were soaked in 50 g of distilled water for 3 h then boiled. Afterward, the formulation was separated from the filtrate, and concentrated to 0.4 g/mL. The solution was stored at 4^°^C and diluted with water when use.

### Animals and treatments

Twenty male SD rats and 20 female SD rats weighing 190 ± 10 g (Jiangsu University, China) were fed with a 45% high-fat and high-sugar diet after 3 days of rest for 6–7 weeks, then received intraperitoneal injection of streptozotocin solution (S6089) at the dose of 35 mg/kg. Rats with fasting blood glucose greater than 11.1 mmol/L were considered as successful models of type 2 diabetes. Afterward, long-term restraint and 2 mL of 1 g/mL *Folium Sennae* decoction gavage two times a day were applied on T2DM rats. Animal models were restrained with wide scotch tape on their shoulder, forelimbs and chest, and restricted from scratching the head and face for 1 h, whereas other activities were allowed.

Modeled rats were divided into five groups as listed: model group, three TXYF groups of low (5 mL/kg), medium (10 mL/kg), and high dosage (15 mL/kg), respectively, and Trimebutine Maleate (TMT) as positive control group, 6 rats in each. A control group that received Weifuan, a control drug, was also established. All rats were treated for 14 days.

The study was approved by the Ethics Committee of Jiangsu University. Approval code is UJS-lACUC-2021070103.

### Model evaluation

The model was evaluated by the changes in body weight, fecal water content, and blood glucose of rats in each group, before, and after treatment, respectively.

### Histological observation

The histopathology of colons was observed by HE method. Fresh tissues were fixed for 18 h in 10% formaldehyde and dehydrated with ethanol solutions, then embedded in paraffin. Four microgram of continuous sections were dewaxed after baking at 58°C for 18 h, then hydrated with all levels of ethanol solutions, stained with hematoxylin for 5 min, fractionated in 1% hydrochloric acid alcohol for 30 s, colored with ammonia, and sealed with resin after ethanol dehydration. All sections were examined and photoed under a microscope for analysis.

### Enzyme-linked immunosorbent assay for the determination of TNF-α, IL1β, IL6, IL10, in colonic tissues

After accurately weighing the animal tissue 9 times the volume of its weight of normal saline, was added to mechanically homogenize a 10% solution under ice-water bath conditions. Next, the homogenate was centrifuged for 10 min at 2,500–3,000 rpm. TNF-α, IL1-β, IL-6, and IL-10 in the supernatant were measured according to the instruction ELISA kit (Thermo Fisher Scientific Invitrogen, USA) of TNF-α, IL1-β, IL-6, and IL-10 to determine the content in the samples.

### Immunohistochemistry for claudin-1

The slices were sequentially placed in xylene I for 15 min, xylene II for 15 min, xylene III for 15 min, anhydrous ethanol I for 5 min, anhydrous ethanol II for 5 min, 85% alcohol for 5 min, 75% alcohol for 5 min, respectively, and washed with distilled water. Afterward, tissue sections were placed in a repair box filled with citric acid antigen retrieval buffer (pH 6.0) and retrieved in a microwave oven at medium heat for 8 min for boiling, then incubation for 8 min, and medium-low heat for 7 min. After natural cooling, the slides were placed in PBS (pH 7.4) and rinsed for 5 min on a destaining shaker for 3 times. Next, prepared sections were incubated in 3% hydrogen peroxide solution, for 25 min in the dark at room temperature then rinsed for 5 min on a destaining shaker for 3 times. In the histochemical circle, 3% BSA or rabbit serum was added to cover the tissue evenly and seal it at room temperature for 30 min. After gently shaken off the blocking solution, slices were dropped with the primary antibody prepared in a certain proportion of PBS and incubate in a wet box at 4°C overnight, then. The slides were washed in PBS (pH 7.4) for 5 min on a destaining shaker for 3 times. Next, when the section was slightly dried, it was dropped a secondary antibody (HRP-labeled) corresponding to the primary antibody was added to cover the tissue, then incubated at room temperature for 50 min. Afterward, slides were washed in PBS (pH 7.4) for 5 min on a destaining shaker for 3 times. And then, freshly prepared DAB solution was applied to develop color under the microscope. After the HE re-staining for about 3 min and washed with running water, the slide was put into solutions in the order of 75% alcohol for 5 min, 85% alcohol for 5 min, anhydrous ethanol I for 5 min, anhydrous ethanol II for 5 min, and xylene I for 5 min, respectively, for dehydration and transparency. Finally, sections were mounted with neutral gum, then examined and photoed with a microscope.

### Pathway enrichment and analysis

Cross-sectional targets were collated and imported to the metascape database^1^ with GO and KEGG analysis to screen out the biological process and related pathways of TXYF in treating D-IBS and T2DM under the condition of *P* < 0.01.

### Western blot analysis

After washing the tissue with PBS to remove blood stains, it was cut into small pieces and homogenized, then applied protein lysis buffer and centrifuged at 12,000 rpm for 10 min. The supernatant was collected. Next, protein solutions were balanced with BCA reagents and added 5X protein loading buffer at the ratio of 4:1, then boiled until denaturation. Afterward, the protein concentration was measured with a BCA protein detection kit, and an equal volume of protein was loaded on 12.5% SDS-PAGE. Whereafter, the surface were blocked with 5% non-fat milk for an hour and incubated with RAGE antibody (GB11278) for 2 h, respectively. Finally, the membranes were rinsed 3 times and incubated with specific secondary antibodies for 1 h, respectively. Visualization of protein signals was detected with an ECL system (Millipore Corporation, Billerica, USA) using equal amounts of BeyoECL A and BeyoECL B. the levels of SOD (Cat# KTB1030) and MDA (Cat # KTB1050) in serum were tested following the instructions (Abbkine). All the SOD and MDA enzyme concentrations were expressed in international units.

### Statistical analysis

Data were expressed as average ± SD. Comparisons between the two groups were determined by a Student’s *t*-test or by One-Way ANOVA (GraphPad software, San Diego CA, USA). A *p*-value less than 0.05 was considered statistically significant.

## Results

### TXYF improves the body weight, blood glucose and fecal water content of D-IBS rats

**FIGURE 1:**
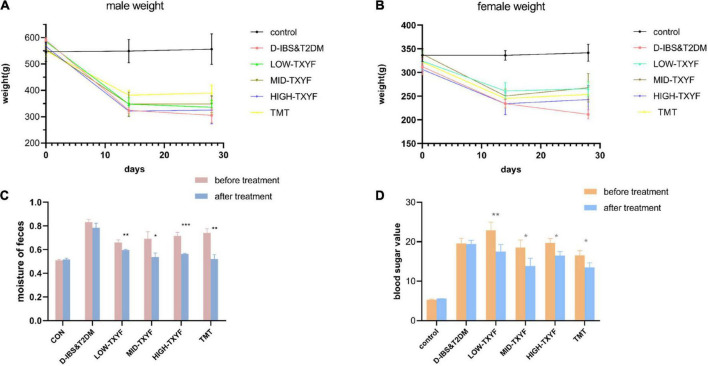
Effect of TXYF on characterization in male D-IBS combined with T2DM rats. Effect of TXYF on weights in male rats **(A)**; effect of TXYF on weights in female rats **(B)**; water content of rat feces **(C)**; blood glucose values in rats **(D)**; data are represented as mean ± S.D. (*n* = 3), *P < 0.05, **P < 0.01, ***P < 0.001 vs. D-IBS&T2DM group.

As showed in Figure 1, the body weight of rats in disease groups was statistically reduced than that in the control group. After the TXYF treatment, their body weight significantly increased. Besides, rats in TXYF groups and the positive control group had significantly lower fecal water content and blood glucose than those in the control group. These results suggest TXYF treatments significantly reversed the weight loss and diarrhea symptoms caused by D-IBS of rats ameliorated their blood glucose.

### Tongxie Yaofang ameliorated the pathological changes in colonic tissues

**FIGURE 2:**
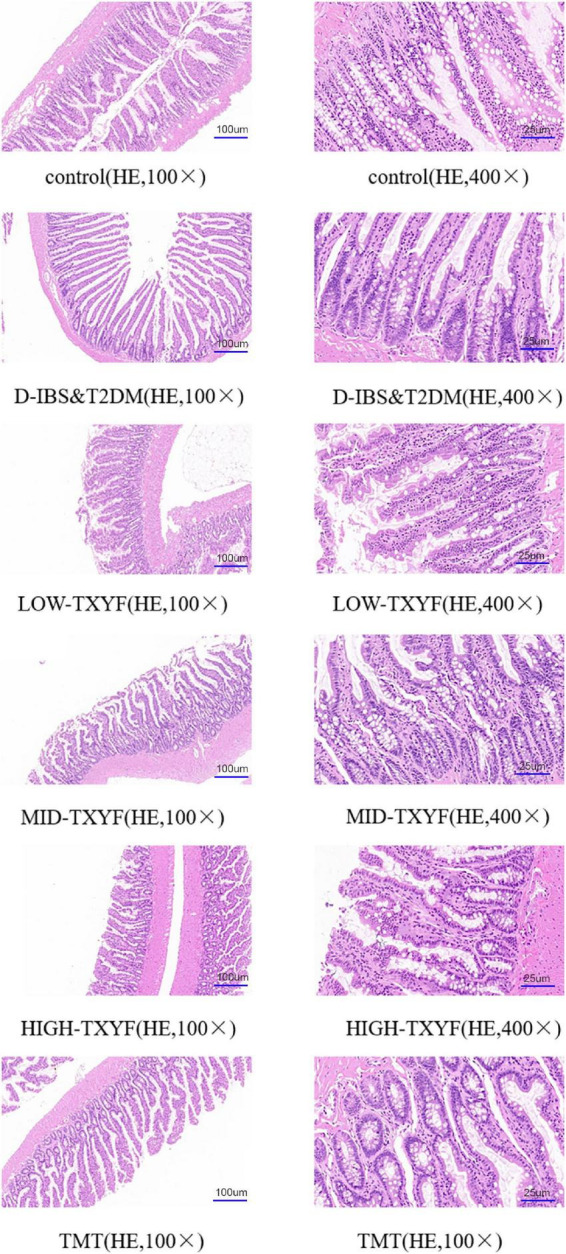
Histopathological examination of colon tissue (100×, 400×, *n* = 3).

As showed in Figure 2, edema, a small amount of inflammatory cell infiltration and hyperplasia in connective tissues were observed in the colon of rats with D-IBS and T2DM. TXYF treatments significantly ameliorated these symptoms, controlled the inflammation and recruited the structure of the colon.

### Tongxie Yaofang regulates the expression of TNF-α, IL-1β, IL-6, and IL-10 in colonic tissues

**FIGURE 3:**
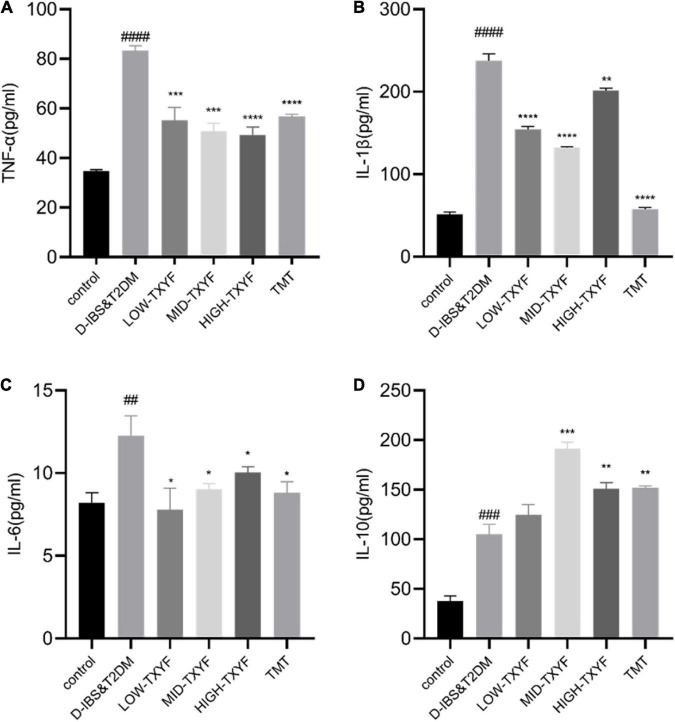
Effects of TXYF on TNF-α, IL-1β, and IL-6 and IL-10 in colonic tissue in rats with D-IBS&T2DM. The expression levels of TNF-α **(A)**, IL-1β **(B)**, IL-6 **(C)** and IL-10 **(D)** in colonic tissue were determined using ELISA (*n* = 6). Values are presented as means ± SD (*n* = 3). ^##^*P* < 0.01, ^###^*P* < 0.001, and ^####^*P* < 0.0001 and ^##^*P* < 0.01 compared with the control group; *P < 0.05 and **P < 0.01 compared with the D-IBS&T2DM group.

Levels of TNF-, IL-1, IL-6, and IL-10 in the tissues in Figure 3 were determined by ELISA. Among them, the expression of TNF-α in disease groups was significantly higher than that in the normal group (*P* < 0.0001). After TXYF administrations, the TNF-α level decreased significantly (*P* < 0.001, Figure 3A). Concentrations of IL-1β and IL-6 in colonic tissues were also quantified by ELISA with consistent results (Figures 3B,C). Besides, the expression of IL-10 was higher in disease groups than that in the control group (*P* < 0.0001, Figure 3D). Hence, TXYF may have helped reducing the chronic inflammation of rats with D-IBS and T2DM induced by *Folium Sennae.*

### Tongxie Yaofang regulated the expression of claudin-1 in colonic tissues

**FIGURE 4:**
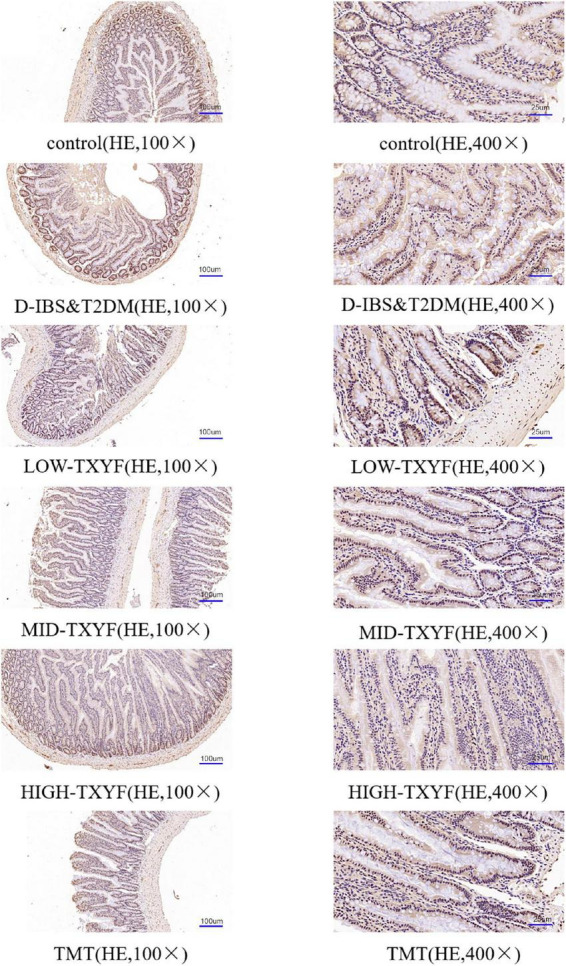
Representative images of claudin-1 in IHC sections (100×, 400×) from colon tissue (*n* = 3).

Claudin-1 levels were measured by immunohistochemistry methods (Figure 4). The expression of claudin-1 in colonic tissues was lower in disease groups than that in the control group. After drug administrations in each TXYF group and positive control drug, TXYF increase the claudin-1 level and effectively ameliorated the change in intestinal permeability induced by *Folium Sennae* in rats with D-IBS and T2DM.

### Potential mechanism of Tongxie Yaofang in treating type 2 diabetes mellitus and irritable bowel syndrome with diarrhea

**FIGURE 5:**
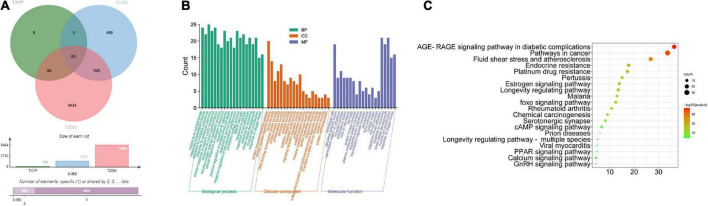
Common targets of TYXF, D-IBS and T2DM **(A)**; list of relevant pathways obtained by GO enrichment and KEGG enrichment **(B,C)**.

As showed in Figure 5A, active ingredients of TXYF effects 65 overlapped targets of D-IBS and T2DM. Afterward, analyzed by GO and KEGG enrichment, the RAGE signal pathway has the highest association with its mechanism (Figures 5B,C).

### Tongxie Yaofang regulated the expression of RAGE proteins

**FIGURE 6:**
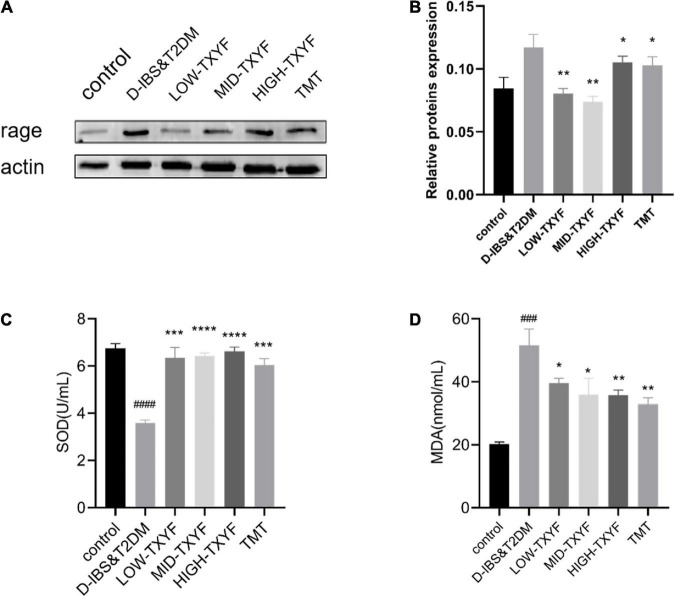
Protein expression level of RAGE was measured using western blotting (*n* = 3) **(A,B)**. Effects of TXYF on SOD and MDA in serum in rats with D-IBS&T2DM. Values are presented as means ± SD (*n* = 3), ^###^*P* < 0.001 and ^####^*P* < 0.0001 compared with the control group; *P < 0.05, **P < 0.01, ***P < 0.001, and ****P < 0.0001 compared with the D-IBS&T2DM group **(C,D)**.

The expression level of RAGE proteins in intestinal mucosa of rats with D-IBS and T2DM in each disease group is shown in Figure 6. The level of RAGE on disease groups was significantly lower than that in the control group (*P* < 0.01). Compared to the model group, RAGE protein expressions in all TXYF groups and the positive control group were significantly higher. As showed in Figures 6C,D, the expression of SOD in disease groups was statistically reduced than that in the control group (*P* < 0.0001). After the TXYF treatment, the SOD level increased. Besides, the expression of MDA in disease groups was significantly higher than that in the normal group (*P* < 0.001). After TXYF administrations, the MDA level decreased significantly. TXYF may alleviate inflammation by regulating RAGE-induced oxidative stress.

## Discussion

This study confirmed the therapeutical effect of TXYF in reducing intestinal inflammation in liver-depression and spleen-deficiency rats with D-IBS and T2DM, and ameliorating the expression of TNF-α, IL-1β, IL-6, and IL-10 in the colon. We also find that TXYF may influence inflammatory factors *via* the RAGE pathway.

There is mounting evidence that IBS and mental health issues are inextricably linked, with dysregulation of gastrointestinal tissues and disruption of gut flora diversity leading to disruption of the gut-central nervous connection and triggering a variety of mental health issues, according to research that has shown that there is growing evidence of this connection (25). 5-hydroxytryptamine (5-HT) plays an important role in intestinal motility, secretion, visceral hypersensitivity and inflammation, and the imbalance of this neurotransmitter and its transporters may lead to IBS (26). Alterations in the intestinal flora caused by probiotics have also been found to have an effect on the expression of associated factors and signaling pathway proteins in the intestinal tissues, this was discovered through research (27). Probiotics have been shown to have an effect on the microflora of the gut, however, the mechanism underlying this effect has not been studied. To sum up, IBS inflammation is the result of a combination of neurological, brain-gut axis, and intestinal flora mediations, all of which merit additional research.

TXYF, originally recorded by Zhu Zhenheng in Danxi Xinfa in Yuan Dynasty, is commonly employed in treating diarrhea, including gastrointestinal discomfort and mental issues as its complications, caused by liver-depression and spleen-deficiency. In modern medicine, TXYF is most frequently prescribed for patients with IBS and chronic enteritis. *Pericarpium Citri Reticulatae* (chenpi) in TXYF may reduce inflammation by acting on certain signaling pathways, such as PI3K/AKT (28). In a study of *Radix Paeoniae Alba* (baishao) and *Rhizoma Atractylodis Macrocephalae* (baizhu), these TCMs may ameliorate the expression of inflammatory factors (29). In addition, a number of research have come to the conclusion that ketones have a high capacity for scavenging free radicals. This is because of the strong reactivity of the hydroxyl groups, which can provide hydrogen and electrons to radicals in order to stabilize them. Zhou et al. discovered that *Atractylodis macrocephala* had the ability to inhibit NF-κB and MAPK, which resulted in a reduction in the inflammatory expression of murine macrophages (30). In addition, a number of research have come to the conclusion that ketones have a high capacity for scavenging free radicals. This is because of the strong reactivity of the hydroxyl groups, which can provide hydrogen and electrons to radicals in order to stabilize them (31). TXYF is rich in ketones, and it hasn’t been found out yet if it has any effect on pathways related to oxidation.

RAGE is a therapeutic target for inflammatory bowel disease (IBD) as it has been demonstrated that RAGE receptors are linked with the activation of immune system in inflammatory responses and mice lacking RAGE are less susceptible to colonic inflammations, as well as RAGE causes inflammation by inducing oxidative stress and its antagonists could effectively threat IBD mice (32) IBD presents an opportunity for RAGE to be used as a therapeutic target.

According to the findings of a number of research projects, the interaction between RAGE receptors and ligands creates a chronic inflammatory pathway that is involved in the mechanism of diabetic complications. Taking all of these factors into account leads one to speculate that blocking RAGE could be an effective therapeutic approach for a variety of diabetic complications (33). Therefore, the rage signaling pathway is expected to be an effective target for the treatment of D-IBS combined with T2DM disease.

## Data availability statement

The raw data supporting the conclusions of this article will be made available by the authors, without undue reservation.

## Ethics statement

This animal study was reviewed and approved by the Institutional Animal Care and Use Committee of Jiangsu University (UJS IACUC).

## Author contributions

WX: conceptualization, methodology, data curation, and writing—original draft. ZZ: methodology, data curation, and writing—original draft. YL: data curation and validation. ML: data curation and investigation. JL: software and validation. WT: writing—original draft, methodology, funding acquisition, and writing—review and editing. All authors agreed to be accountable for all aspects of work ensuring integrity and accuracy.

## Footnotes

1
https://metascape.org/gp/index.html
